# Night Sister

**Published:** 1919-01-18

**Authors:** 


### SOME HOSPITAL TYPES OF BRITISH NURSING.

### VI.—Night Sister.

SHE is not going to be night sister any more, and we are all very sorry indeed. She has been so good to us. When we, as very new probationers, came stumbling into breakfast those first nerve-filled mornings, conscious that our aprons were very new and our strings were all awry, she was always waiting to see we got safely into our seats, and with her wonderful understanding smoothed away the first awkwardness for us. The very junior nurses (after the first few mornings) served round the breakfasts, and when they had finished serving Sister made them sit down while she waited on them. Before she sat down at the head of the "staff" table she made sure that all the probationers had their share of breakfast. She was so good to us. Only once did we see her a little bit vexed with one of "us." A place higher up the table had been missed, probably by accident, and the junior places were all served. Sister looked round the table and discovered the breakfastless one. She walked down to the "pro.s" who had been serving and asked how this had come about. There was no reply, and her remark is still in my mind: "You must serve the others first. You yourselves are last. That is your privilege." She was very patient with us, and always anxious to smooth an awkward moment. When on night duty, running about as extra, if we were told to do something we did not know how to do; if there was "something on" and Sister was about, she was beside us in a moment doing the thing herself, and yet almost making us believe we had *really* done it. She never made us by any chance feel foolish. If there was something happening she thought we were too junior to see, we were out from behind the screens in a moment, and sent away on an errand (for her) which would occupy some little time. She was very keen on the minimum amount of noise through the night. As luck would have it, if I ever happened to drop anything in the kitchen Sister was sure to be passing, and her head was sure to pop round the door, and "You'll be as quiet as you can, won't you, nurse?" would fall on my expectant ears. One night one of us was ill and unable to go on duty. When night sister came up the "pro." was very anxious to go on, but Sister was obdurate. This is what she said: "You are all right here, nurse. No, you need not get up and come on duty. You just go to sleep, and you'll be all right in the morning." And presently a hot-water bottle and a glass of hot milk came up the stairs. We are only junior probationers, "our crowd," but we shall always remember our first night sister, who counted it a privilege to "serve."

